# Project FIT: Rationale, design and baseline characteristics of a school- and community-based intervention to address physical activity and healthy eating among low-income elementary school children

**DOI:** 10.1186/1471-2458-11-607

**Published:** 2011-07-29

**Authors:** Joey C Eisenmann, Katherine Alaimo, Karin Pfeiffer, Hye-Jin Paek, Joseph J Carlson, Heather Hayes, Tracy Thompson, Deanne Kelleher, Hyun J Oh, Julie Orth, Sue Randall, Kellie Mayfield, Denise Holmes

**Affiliations:** 1 Department of Kinesiology, Michigan State University, East Lansing, MI, USA; 2 The Healthy Weight Center, Helen DeVos Children's Hospital, Grand Rapids, MI, USA; 3 Department of Food Science and Human Nutrition, Michigan State University, East Lansing, MI, USA; 4 Department of Advertising, Public Relations, and Retailing, Michigan State University, East Lansing, MI, USA; 5 Division of Sports and Cardiovascular Nutrition, Michigan State University, East Lansing, MI, USA; 6 Institute for Health Care Studies, Michigan State University, East Lansing, MI, USA

**Keywords:** obesity, school intervention, exercise, nutrition, social marketing

## Abstract

**Background:**

This paper describes Project FIT, a collaboration between the public school system, local health systems, physicians, neighborhood associations, businesses, faith-based leaders, community agencies and university researchers to develop a multi-faceted approach to promote physical activity and healthy eating toward the general goal of preventing and reducing childhood obesity among children in Grand Rapids, MI, USA.

**Methods/design:**

There are four overall components to Project FIT: school, community, social marketing, and school staff wellness - all that focus on: 1) increasing access to safe and affordable physical activity and nutrition education opportunities in the schools and surrounding neighborhoods; 2) improving the affordability and availability of nutritious food in the neighborhoods surrounding the schools; 3) improving the knowledge, self-efficacy, attitudes and behaviors regarding nutrition and physical activity among school staff, parents and students; 4) impacting the 'culture' of the schools and neighborhoods to incorporate healthful values; and 5) encouraging dialogue among all community partners to leverage existing programs and introduce new ones.

**Discussion:**

At baseline, there was generally low physical activity (70% do not meet recommendation of 60 minutes per day), excessive screen time (75% do not meet recommendation of < 2 hours per day), and low intake of vegetables and whole grains and high intake of sugar-sweetened beverages, French fries and chips and desserts as well as a high prevalence of overweight and obesity (48.5% including 6% with severe obesity) among low income, primarily Hispanic and African American 3^rd^-5^th ^grade children (n = 403).

**Trial registration:**

ClinicalTrials.gov NCT01385046

## Background

Poor nutrition, physical inactivity and obesity are considered the most pressing health issues in pediatrics and child health. About 75% of U.S. students do not consume 5 or more fruits and vegetables per day [1] and about 60% of children ages 6-11 years do not obtain the recommended 60 min per day of physical activity [2]. While obesity rates have increased across all segments of the population, they have increased most drastically (and are the highest point estimates) among low-income and Hispanic and Black children [3]. Thus, there is an important need to focus intervention on these groups of children [4]; however, few intervention studies have focused on low-income and/or minority children [5-10].

In an effort to improve key behaviors, knowledge, self-efficacy, and attitudes regarding physical activity and nutrition, we developed and will implement an intervention targeted at schools and neighborhoods with predominantly Hispanic and/or Black low-income students (95% of students qualifying for free and reduced lunch). This paper presents an overview of Project FIT, a community- and school-based intervention. The study design, implementation, and preliminary baseline results are explained within this paper.

## Methods/Design

### What Is Project Fit?

The overall objective of Project FIT was to increase physical activity and improve healthy eating among the school children of four urban, low-income elementary schools in Grand Rapids, MI. The project aimed to accomplish this objective by: 1) increasing access to safe and affordable physical activity and nutrition education opportunities in the schools and in the surrounding neighborhoods; 2) improving the affordability and availability of nutritious food in the neighborhoods surrounding the schools; 3) improving the knowledge, self-efficacy, attitudes and behaviors regarding physical activity and nutrition among school staff, parents and students; 4) impacting the 'culture' of the schools and neighborhoods to incorporate healthful values; and 5) encouraging dialogue among community partners to leverage existing programs and introduce new ones. The overall project is comprised of four components including: school, community, social marketing, and school staff wellness.

Project FIT built upon the many strengths and assets that currently exist within the Grand Rapids Public School District and the Grand Rapids community including (but not limited to): innovative school nutrition services, comprehensive school nursing programs, solid academic programming, and strong existing partnerships between the school system and community-based organizations. In addition, an advisory board was established and will meet quarterly throughout the project. A list of the advisory committee members and project partners is provided in Table 1.

**Table 1:** Project FIT Advisory Committee Members and Project Partners.

**Organization**
YMCA of Greater Grand Rapids
Grand Rapid African American Health Institute
Neighborhood Ventures, Inc
Kent Count-Michigan State University Extension
Grand Rapids Chamber of Commerce
Kent County Coordinated School Health
Friends of Grand Rapids Parks
Hispanic Center
Grand Valley State University Kirkhof College of Nursing
Grand Valley State University Community Research Institute
Grand Rapids Public Schools
Lighthouse Communities
True Light Baptist Church
Spectrum Health/Helen DeVos Children's Hospital
Metro Health

### Theoretical Framework

Project FIT is guided by the social-ecological model [11] as a theoretical framework and social marketing principles [12] as a strategic tool. The social ecological model underscores the importance of multiple levels of influence on individual behavior. Additionally, components of the social cognitive theory [12] were implemented to promote the children's attitudes and self-efficacy for making desirable physical activity and nutrition choices. Social marketing refers to the use of (commercial) marketing techniques to design, manage, and implement programs to promote socially beneficial changes in behavior [12,13]. With a particular focus on branding and promotion, various promotional activities were combined with physical activity and nutrition programs to remind and motivate participants to engage in the promoted behaviors. The project was branded as Project FIT, a program that cultivates and reinforces individuals' physical activity and healthy eating.

### Setting And Participants

General characteristics of the schools and participants are shown in Table 2. Five elementary schools located in the Grand Rapids school district were selected by the superintendent to be part of the study (four intervention and 1 control). All schools reported greater than 95% of students qualifying for the Free or Reduced Lunch Program. Child-specific assessments were made on students in grades 3-5. A total of 434 third to fifth grade children were enrolled in the study out of 768 possible (57% overall participation rate; 54% in the intervention schools and 67% in the control school). Third and fourth graders at each school will be followed over the two-year period (Fall 2009 - Spring 2011) to determine the project's impact. The primary parental language (e.g., spoken at home) for the intervention and control schools was as follows: English, 37.6% and 48.6%; Spanish, 19.0% and 38.5%; Other, 1.0% and 0%; did not report, 41.5% and 12.8%, respectively. To have sufficient power to detect a difference of 1000 steps/day (standard deviation 2500 steps/day) or 2 fruit and vegetable servings per week (standard deviation 5 servings per week) or 5 hours of screen time per week (standard deviation 12 hours per week) between intervention and control group with power 0.80 and alpha 0.05, a minimum of 200 subjects were needed. Parental consent and child assent were obtained prior to data collection. All aspects of the study protocol were approved by the Grand Rapids Public School District's and Michigan State University's Institutional Review Boards. Students who returned their completed forms were given a $10 gift card for their families regardless of whether they decided to participate in the study. Parent survey and focus group participants were asked to sign a written consent form prior to participation. For the community component, the target communities were defined as the attendance areas of the four intervention schools plus 400 meters (approximately 0.25 miles).

**Table 2:** Demographic characteristics of the schools and study population, Fall 2009.

	Control	Intervention				Total
		A	B	C	D	
School Enrollment (PK-5)	435	456	235	462	362	1950
Total Eligible Sample (3-5)	170	174	87	181	156	768
Sample Size	114	85	60	86	89	434
% Response	67%	49%	69%	48%	57%	57%
% Free-Reduced Lunch	98.0%	95.3%	97.8%	96.5%	96.7%	96.9%

### Intervention Programs

Representatives from the Grand Rapids Public School District, the YMCA of Greater Grand Rapids, Michigan State University (MSU) and MSU Extension, Blue Cross Blue Shield of Michigan and various community entities worked together to design the intervention (Table 1). The underlying premises of the intervention included sustainability, replicability, dissemination to other Grand Rapids schools and other Michigan school districts, measurable objectives, and informing policy. The intervention will be composed of a series of inter-related components in three general categories of school, social marketing, and community. Each of these components is described in the following text.

### School component

#### 1. School staff training

School personnel training will be provided to assist with the implementation of the intervention components. This training utilizes and builds upon the *Healthier Schools, Healthy Students *course developed by Kent County Coordinated School Health http://kccshp.weebly.com/. During the first year of the intervention, all trainings will be mandatory and part of professional development and staff meeting times for all teachers. During the second year, a combination of mandatory and voluntary evening training sessions will be provided. Training sessions will focus on basic nutrition and physical activity knowledge, fostering healthy behaviors, creating a culture of health and wellness in the schools, and giving specific tips to teachers on how to provide nutrition education and physical activity to their students in the classroom. Monthly newsletters will also be distributed to provide additional nutrition education and physical activity information for educators and ideas for activities and education the teachers could implement in their classrooms.

#### 2. Enhancement of school physical activity and nutrition education

At each intervention school, school staff will receive training and resources to promote physical activity and nutrition education in the classroom. Teachers will be encouraged to provide 30 minutes of physical activity per day and 20 hours of nutrition education per year. Resource distribution will be tailored to the interests at each school. Options for obtaining the 30 minutes of structured daily physical activity include, but are not limited to: physical education class, curricular lessons, dance party in the classroom, use of activity videos/DVDs/Workouts on Demand, and use of active video games (such as Dance Dance Revolution). Options for obtaining 20 hours of nutrition education include, but are not limited to: curricular lessons, Health through Literacy http://www.pe-nut.org/health-through-literacy, and Healthier Classrooms/Healthier Kids electronic toolkit on a flashdrive.

An analysis of sample menus provided by the Grand Rapids Public School District indicated that the district was currently meeting or exceeding most components of the Michigan State Board of Education Nutrition Standards. However, providing healthy foods to students does not ensure that they select or consume those foods. A significant missing piece is nutrition education and healthy food promotion to encourage students to consume the healthy foods offered in the cafeteria and to make healthy selections outside of school. Thus, in addition to classroom nutrition education, each school will launch a program (in collaboration with the school district) that will place "Healthy Eating Coaches" in the cafeteria who will eat a meal with the students and encourage them to try the healthy foods on their plates, including fruits and vegetables. Healthy Eating Coaches will be professional-level teaching staff (primarily teachers) trained by nutrition services to encourage students to taste and eat healthy foods offered for lunch.

#### 3. Improvements to nutrition and physical activity policies and environments through a Coordinated School Health Team

The state of Michigan is unique in terms of the comprehensive tools available to assist schools in achieving healthy policies and environments for nutrition and physical activity http://www.mihealthtools.org/healthyschools.asp. Developed in collaboration by the Michigan Department of Community Health, the Michigan Action for Healthy Kids Coalition, and Michigan Department of Education Team Nutrition, the online tool set, Healthy School Action Tools (HSAT), has been utilized by over 1,000 schools to date. Project FIT schools will be required to form a Coordinated School Health Team, complete the HSAT healthy eating & nutrition and physical activity & physical education topic questions, develop an action plan and implement at least three food/nutrition or physical activity policy or environmental improvements during the course of the project. Funding will be provided to Coordinated School Health Teams in order to implement their action plans. Suggested ideas for action plans include providing healthy food options for school stores, classroom parties, and fundraisers; improving playground equipment, and elimination of use of food as a reward.

#### 4. School staff wellness

Since teachers and staff members can play a significant role in the success of the development of a healthy school culture, wellness opportunities for the school staff will be pursued as part of Project FIT. Teachers and staff members will be encouraged to be physically active and eat healthy foods with students through the school-based portion of this project. In addition to promoting confidence in championing healthy eating and physical activity throughout the school day, the staff training and newsletters are intended to focus on staff wellness by increasing knowledge and self-efficacy in their own health behaviors. Opportunities such as evening zumba classes, mileage/walking clubs, and pedometer challenges will be also provided to teachers and staff.

### Social marketing component

The social marketing component of Project FIT focuses on branding and promotion strategies. For branding, a Project FIT logo was professionally produced and will be used across all the Project FIT programs, events, and promotional items to integrate all the different components into one project. In order to promote Project FIT, items related to physical activity and healthy eating will be produced (e.g., water bottle, grocery bag, pedometer, and jump rope). Each of the items will also include a short message that encourages physical activity and healthy eating. These items will be distributed at the participating schools' open houses, parent-teacher conferences, and parent-student night events. They will also be distributed to the neighborhood health clinics, churches, and community centers. A series of FIT stickers will be produced and distributed by Healthy Eating Coaches to students who try new healthy foods during school mealtime so that students are motivated to try more new healthy foods to collect different kinds of stickers. In addition, selective mini-media such as school newsletters, brochures, posters, and calendars will be used for communication and education. In particular, a calendar will be developed as an educational medium to inform parents about types of physical activities, healthy cooking recipes, and community events.

### Community-based component

The community component will complement the school component and affect those children who attend the participating schools and their families. Additionally, the community intervention will be planned to work within the unique character of the existing greater community, and therefore, goals for the intervention include that it be non-redundant and complementary to existing programs, highly collaborative, and additive/synergistic with other project components.

To understand the resources available within the neighborhoods surrounding the four intervention schools, a community assessment was performed in collaboration with the Grand Valley State University (GVSU) Community Research Institute as formative evaluation. This included: a) interviews to learn of existing programs and organizations that work directly or indirectly on childhood obesity within Grand Rapids, b) development of a list of stores offering food within the target areas by combining lists of all stores within Grand Rapids that accept bridge cards, purchased list of food stores, and lists from telephone directories of businesses described as a market or grocery, c) Nutrition Environment Measures Survey (NEMS) of the consenting stores within the target area, d) geographical information system (GIS) mapping of green space and parks within the target area, as well as other community resources such as congregations, pantries and food banks, e) focus groups of community members, and f) neighborhood demographics including: population density, race/ethnicity, sex, poverty level, age, and percentage of children qualifying for the Free or Reduced Lunch program.

Four community-related intervention components will include the following: 1) Community Wellness Events: with the YMCA of Greater Grand Rapids, a series of events to introduce opportunities for nutrition education and physical activity; 2) Enhancement of After-school Programming: with the GVSU Kirkhof College of Nursing, after-school programming provided by the Grand Rapids Parks & Recreation will be evaluated, a new curriculum recommended, implementation and evaluation plans developed, and implementation started; 3) Healthy Corner Stores: With Neighborhood Ventures, Inc., a healthy corner store program modeled after The Food Trust's work [14] will be developed, implemented, and named FIT Stores; 4) Enhancement of Parks: with Friends of Grand Rapids Parks, area parks will be improved to encourage increased physical activity of children and residents.

### Evaluation Methods

The evaluation of the project will include the following measures: child-specific outcome measures; school environment, policies and programs; process and formative evaluation; parent survey, and community focus groups. Each of these measures is described in the following text and represents baseline data collection.

### School Level

#### Child-specific outcomes

Student measures including physical measures and questionnaires occurred during Fall 2009, and follow-up measures will occur in Spring 2010, Fall 2010 and Spring 2011. Measures will be/were completed on consenting 3^rd^, 4^th ^and 5^th ^graders, unless otherwise specified.

*Habitual free-living physical activity *was determined by self-report and also objectively measured by pedometer. The self-report question was the same used in the Youth Risk Behavior Survey asking how many days per week children engaged in moderate-to-vigorous intensity physical activity. Subjects were also instructed to wear a pedometer (Digiwalker 200-SW) for a 1-week period. In a sub-sample of subjects, habitual free-living energy expenditure and physical activity was assessed with the Sensewear Pro 3 Armband (SWA). The SWA is a wireless, non-invasive, multi-sensor activity monitor that is worn over the triceps muscle. The monitor integrates data from 5 sensors to estimate energy expenditure under free-living conditions [15].

##### Screen time

Children's weekly amount of time viewing television, playing video games, and online computer use was self-reported by the children. Children were asked to indicate the number of hours they watch/play on weekdays and weekends for each of the screen media.

##### Nutrition and physical activity knowledge, attitudes, self-efficacy, and behavior

Two surveys including questions about nutrition and physical activity knowledge, attitudes, behaviors and self-efficacy toward these behaviors were administered by program staff. The surveys were completed by the children under the guidance of staff trained to administer the surveys in a standardized manner in a group classroom setting. Food frequency, knowledge and positive outcomes questions were adapted from the School Physical Activity and Nutrition Questionnaire [16] and the 4^th ^grade Nutrition Education Survey [17]. The self-efficacy questions for nutrition and physical activity behaviors were adapted from a tool designed for youth athletes [18] and used a five-point self-efficacy scale with circles ranging from small to large to allow children to report their level of confidence for a specific behavior [19]. Additionally, 5^th ^grade students completed the Block Food Frequency Questionnaire (FFQ) for Kids (Block Dietary Data Systems, Berkeley, CA; http://www.nutritionquest.com). The FFQ report includes daily estimates of macro-nutrients, major micro-nutrients, and servings per day of key food groups and has been validated against a 24-hour dietary recall (i.e., no significant differences were observed in estimated energy, protein, fat and saturated fat intakes between the FFQ and one 24-hour recall).

##### Plate consumption assessment

Plate consumption (PC) was conducted in participating schools for 3 consecutive days and included an evaluation of 125 third to fifth grade student's trays per school. The PC method (note: also referred to as plate waste) includes quantifying the amount of all food and beverage consumed by students during the lunch period using a visual-photographic evaluation of all food and beverages remaining on the plate using standardized procedures established by Williamson [20]. The PC of each food item on participating student's tray is documented by the amount consumed from none (0), 1/4 (0.25), 1/2 (0.5), 3/4 (0.75), or all (1.0) in reference to foods on a standardized reference tray. Volume of milk remaining on the tray was measured (in ml) and subtracted by the amount in the standard carton (240 ml) to determine consumption. During the monitoring period, the cafeteria personnel followed their usual standardized procedures including providing standardized portions of each menu item to all students who purchase school lunch and those who qualify for free lunch. Participating student's trays were tagged with an identification number. When students completed their lunches, their trays were taken to a designated area where research staff completed the photographic assessment and the volume of milk consumed from each tray. The visual analyses of photos were performed in duplicate by two trained research assistants blinded to each other. For the 21% of reviews that did not match, a third non-blind review was conducted by an additional evaluator which was used to make a final decision. Due to technical difficulties at the school, plate consumption for the control school was not conducted.

##### Physical measures

The physical measurement battery includes: body mass index (BMI), body composition (% body fat via bioelectrical impedance), waist circumference, and resting blood pressure. Stature and body mass were measured according to standard procedures [21]. The body mass index (BMI) was calculated from the equation: BMI = body mass (kg)/stature (m^2^). BMI is mapped into a percentile using age- and sex-specific reference values of the CDC growth charts to determine weight status (e.g., underweight < 5^th ^centile; normal weight 5-84^th ^centile; overweight > 85-94^th ^centile; obese > 95th centile) [22]. Waist circumference was measured as a proxy for abdominal or visceral adiposity using a Gullick tape to the nearest 0.1 mm at the level of the umbilicus. Body fatness is measured using a foot-to-foot bioelectric impedance device (Tanita Corporation, Tokyo, Japan). Resting blood pressure is measured manually following standardized procedures [23]. Prior to data collection, all technicians were trained by JCE.

#### School environment, policies and programs

Personnel from each of the participating schools completed the School Environment and Policy Survey [24]. The survey consists of three modules designed to capture nutrition and physical activity environments, policies and programs offered in the schools.

#### Process and Formative Evaluation of the school component

A process/program evaluation will be conducted throughout the study to determine how dose delivered and fidelity to each component of the intervention relates to student outcomes. School and/or study personnel will be asked to use checklists to record delivery of physical activity opportunities, nutrition lessons, and any modifications and/or feedback they have regarding lessons. Attendance will be taken at all activities. Occasionally, project staff will randomly observe selected lessons at each intervention school to assess fidelity. Physical education teachers, classroom teachers, and principals will also be queried about the school environment during interviews (post-intervention); this includes documenting changes at control schools via interviews. All interviews will be conducted according to standardized procedures, and an appropriate informed consent process will take place prior to conducting them.

### Social Marketing

Social marketing activities in the schools targeting children will be evaluated using the paper-and-pencil survey questionnaires that include physical activity and knowledge, attitudes, self-efficacy, and behavior (described above). The questions include brand recall (e.g., have you heard about Project FIT?), mode and channel of media (e.g., where have you heard about it?), brand knowledge (e.g., what is Project FIT?), brand attitudes, and brand loyalty (i.e., commitment to project FIT). These questions will be drawn from the social marketing literature [25,26]. In addition, branding and promotion materials that are distributed will be counted as part of output/process measures. The teacher survey, as part of process evaluation for the school component, will also ask teachers of how useful each of the promotion materials would be to motivate students to engage in the promoted behaviors.

Formative evaluation research was conducted among parents (n = 286) of the students in the participating schools. The purpose of the evaluation research was to understand 1) their current physical activity with their children and eating patterns at home, 2) the perceived barriers for them to engage in physical activity and health eating, and 3) kinds of information related to physical activity and healthy eating that they prefer. The parents were recruited at the teacher-parent conferences or other school events.

A follow-up parent survey will be conducted among parents of the students in the participating schools during the program. The purpose of the follow-up research is to examine any change or improvement of 1) physical activity with their children and healthy eating patterns at home, 2) knowledge about physical activity and nutrition, and 3) parents' awareness of, attitude toward, and commitment to Project FIT.

### Community Assessment

The community assessment included the following methodologies--a survey of food stores using the NEMS tool [27], mapping of store results with other community assets, focus groups with neighborhood residents, and secondary analysis of parent surveys (described above). The various methods were selected to reinforce the main component of the community assessment--the NEMS survey. Focus groups were also conducted to allow reinforcement of other findings.

#### NEMS - Identification of Stores

A store list was generated by combining the following: a U.S. Department of Agriculture list of the names and addresses of all stores in greater Grand Rapids that accept food stamps, a compiled a list of all businesses in the Grand Rapids area described as a "market", "convenience store" or "grocery", and a purchased list of area stores from a commercial provider. The final store list was loaded into the GIS mapping system to determine those stores within the communities to be assessed. This process yielded 44 stores.

#### NEMS - Surveying of Stores

A nutritionist trained students on the procedures involved in administering the NEMS tool and the student research assistants implemented the NEMS at the 23 consenting stores. The NEMS tool was modified to include a cereal category and to include points for stores offering more varieties of foods. To provide some comparative NEMS scores for small stores found in the community areas, one large grocery was identified located within one mile of each of the four community areas. A research assistant surveyed each of these four stores in May 2010. Six stores were surveyed twice to determine the reliability of data collection methodology.

#### Community Scan and Focus Groups

Community assets including religious congregations, key and support food pantries, and public parks were identified and mapped within each of the neighborhoods around the four intervention schools. Focus groups were held with community members from each of the four school areas.

#### Process Evaluation

Process evaluation will occur for each community component. Regarding healthy stores, the FIT Store program will utilize the NEMS done for participating stores during the community assessment as a baseline measurement. In addition, photographs of the store taken from inside and outside the store, interviews with the store owner, a customer survey, and a more complete stock assessment form created by The Food Trust for their Healthy Corner Store Program will be utilized for baseline measurements. Follow-up measures will include items such as a repeat of the NEMS, The Food Trust stock assessment, photographs, and customer survey.

Various measures such as the use of the System for Observing Physical Activity and Recreation in communities (SOPARC) tool [28] will be used to assess the improvement of parks. Regarding the after-school program, various health-related assessments such as height, weight, and physical activity will take place. Program components will also be evaluated.

## Results

The preceding section outlined the methodology for all data collection throughout the project. Given the purpose of this paper and the restrictions of space, we only focus on selected results at baseline. Subsequent reports will detail all outcomes of this project.

### Child physical activity, nutrition and weight status

Table 3 provides baseline results of the physical characteristics, physical activity, screen time, and dietary habits of the total sample and by control and intervention schools grouped by sex. Self-reported habitual physical activity was similar between control and intervention schools (3.7 ± 2.6 days vs. 4.0 ± 2.5 days, respectively). About 30% of students at the control and intervention schools met the physical activity recommendation of 60 min/day and weekend screen time was 5.3 ± 3.1 hours per day for the total sample. The percentage of students viewing ≥2 hours per day of total screen time was about 75% for both the control and intervention schools Self-reported diet frequency (times consumed yesterday) was also similar between control and intervention schools. Overall the students reported frequency (times consumed yesterday) of consuming various foods and beverages included: vegetables 1.0, fruit 1.4, fruit juice 1.0, cereals 0.60, beans 0.40, French fries and chips 0.90, sugar drinks 1.6 and desserts and candy 2.0 times on the day prior to the survey. According to the PC analysis, intervention school students consumed on average 73% of their entrée, 61% of their fruit, 36% of their vegetable, 63% of their grain, and approximately 70 ml of 240 ml or 29% of their milk at lunch.

**Table 3:** Physical characteristics, physical activity, screen time, and dietary measures of the total sample and by control and intervention schools grouped by sex.

	Control School			Intervention Schools			TOTAL
	Males n = 47	Females n = 59	Total n = 106	Males n = 139	Females n = 158	Total n = 297	n = 403
Age (yrs)	9.6 (0.9)	9.7 (1.0)	9.7 (0.9)	9.6 (0.9)	9.6 (1.0)	9.6 (0.9)	9.7 (0.9)
BMI percentile	77.5 (24.7)	70.0 (29.0)	73.2 (27.3)	74.3 (24.7)	71.2 (28.8)	72.6 (26.9)	73.0 (27.0)
% Underweight	0%	0%	0%	0%	1.3%	0.7%	0.5%
% Normal weight	46.8%	55.9%	51.9%	50.7%	50.6%	50.7%	51.0%
% Overweight	19.1%	11.9%	15.1%	17.4%	19.0%	18.2%	17.4%
% Obese	25.5%	28.8%	27.4%	26.1%	22.8%	24.3%	25.1%
% Severely Obese	8.5%	3.4%	5.7%	5.8%	6.3%	6.1%	6.0%
***Physical Activity***							
Self-report physical activity (d/wk)	4.3 (2.7)	3.2 (2.5)	3.7 (2.6)	3.9 (2.6)	4.0 (2.5)	4.0 (2.5)	3.9 (2.6)
% reporting ≥5 days	48.8%	33.3%	40.0%	46.4%	45.0%	45.7%	43.7%
% reporting 7 days	39.5%	21.1%	29.0%	31.0%	30.0%	30.4%	29.9%
***Screen Time***							
Weekday TV (hrs/d)	1.4 (1.4)	1.7 (1.5)	1.6 (1.4)	1.9 (1.5)	1.8 (1.4)	1.8 (1.5)	1.7 (1.5)
Weekend TV (hrs/d)	2.2 (1.3)	2.4 (1.3)	2.3 (1.3)	2.4 (1.4)	2.4 (1.4)	2.4 (1.4)	2.4 (1.4)
Weekly TV (hrs/d)	1.7 (1.2)	1.9 (1.3)	1.8 (1.3)	2.0 (1.4)	2.0 (1.3)	2.0 (1.3)	1.9 (1.3)
Weekday Screen Time (hrs/d)	3.7 (3.0)	3.4 (3.0)	3.5 (3.0)	5.1 (3.4)	3.9 (3.4)	4.4 (3.4)	4.1 (3.3)
Weekend Screen Time (hrs/d)	5.6 (3.1)	4.7 (3.0)	5.1 (3.1)	6.0 (3.0)	4.9(3.1)	5.4 (3.1)	5.3 (3.1)
Total Screen Time (hrs/d)	4.3 (2.8)	3.8 (2.8)	4.0 (2.8)	5.4 (3.2)	4.2 (3.2)	4.7 (3.2)	4.5 (3.1)
% ≥ 2 hrs/d screen time	74.4%	73.1%	73.7%	82.1%	71.8%	76.7%	75.6%
***Diet***							
Whole grain breads (times/d yesterday)	0.50 (0.77)	0.41 (0.56)	0.45 (0.66)	0.67 (0.79)	0.59 (0.72)	0.62 (0.75)	0.58 (0.73)
Cereals (whole grain) (times/d yesterday)	0.72 (0.88)	0.37 (0.49)	0.52 (0.70)	0.70 (0.94)	0.55 (0.78)	0.62 (0.86)	0.60 (0.82)
French fries or chips (times/d yesterday)	1.00 (1.12)	0.85 (0.87)	0.91 (0.98)	1.00 (0.99)	0.88 (0.87)	0.93 (0.93)	0.93 (0.94)
Vegetables (times/d yesterday)	1.18 (1.15)	0.93 (0.81)	1.04 (0.97)	1.09 (1.03)	0.90 (0.92)	0.99 (0.98)	1.00 (0.97)
Beans (times/d yesterday)	0.36 (0.61)	0.19 (0.39)	0.26 (0.50)	0.51 (0.79)	0.41 (0.68)	0.46 (0.74)	0.41 (0.69)
Fruit (times/d yesterday)	1.57 (1.09)	1.61 (1.00)	1.59 (1.03)	1.42 (1.05)	1.22 (0.92)	1.31 (0.99)	1.38 (1.01)
Fruit juice (times/d yesterday)	0.91 (0.96)	1.07 (0.91)	1.00 (0.93)	1.05 (0.97)	0.96 (0.89)	1.00 (0.93)	1.00 (0.93)
Dairy beverages and food* (times/d yesterday)	3.3 (2.2)	2.8 (1.4)	3.0 (1.8)	3.2 (2.0)	2.6 (1.7)	2.9 (1.8)	2.9 (1.8)
Sugar Drinks** (times/d yesterday)	1.9 (1.3)	1.4 (1.1)	1.6 (1.2)	1.9 (1.6)	1.4 (1.5)	1.6 (1.6)	1.6 (1.5)
Desserts and Candy*** (times/d yesterday)	2.0 (2.1)	1.3 (1.6)	1.6 (1.8)	2.1 (2.2)	2.1 (2.0)	2.1 (2.1)	2.0 (2.0)
***Plate Consumption#***							
Entrée				0.75 (0.25)	0.72 (0.27)	0.73 (0.26)	
Fruit				0.55 (0.33)	0.65 (0.29)	0.61 (0.31)	
Vegetable				0.35 (0.32)	0.37 (0.32)	0.36 (0.32)	
Grain				0.65 (0.31)	0.61 (0.31)	0.63 (0.31)	
Milk (ml)				58.6 (68.9)	79.3 (73.4)	69.8 (71.9)	

Diet measures expressed as times eaten yesterday. Values are mean (standard deviation) or percentage

*Summary variable combining 3 SPAN items: cheese, milk and yogurt

**Summary variable combining 2 SPAN items: sugar drinks and pop

***Summary variable combining 3 SPAN items: desserts, frozen desserts and chocolate candy

#proportion of item consumed. Note: data from control school was lost due to technical difficulties.

The mean BMI percentile for the total sample was 73 ± 27 percentile. Of the total sample, 17.4% were classified as overweight and 25.1% as obese. Furthermore, 6.0% of the total sample were severely obese (BMI ≥99^th ^percentile).

### Parent survey

About half of the parents reported performing amount of a recommended  mount of aerobic activity every week (53.1%)--i.e., a total of 150 minutes of moderate-intensity aerobic activity (e.g., brisk walking) every week or 75 minutes of vigorous-intensity aerobic activity (e.g., jogging or running)--and muscle-strengthening activities on 2 or more days a week (48.1%). About 50% of the parents reported doing physical activity with their children about 1-2 times a week, followed by almost none (27.0%), about 3-4 times (11.5%), and almost everyday (11.9%). About 90% reported a daily consumption of fruit and vegetables less than 5 servings despite 53.1% reporting that they were satisfied with their current fruits and vegetable consumption.

With regards to barriers to healthy habits, respondents reported that people do not eat healthy foods because they do not have enough time (25.5%), do not know how to prepare healthy food (24.7%), it is perceived as unimportant (23.4%), or too expensive to buy healthy food (19.9%). About a half of parents reported that people are not physically active because people do not have enough time (48.3%). The parents also reported that physical activity is perceived as unimportant to other people (16.7%) and there is not enough space or place for exercise (12.4%).

The largest proportion of parents wanted to learn from Project FIT how to make healthy food (43.6%), followed by places to get inexpensive and healthy food (28.7%). Parents were also interested in knowing the appropriate frequency and duration of exercise (32.3%) and types of exercise that are good for health (29.9%). Among possible communication channels, parents wanted to receive physical activity and nutrition messages from the school newsletter (22.7%), brochure (20.5%), website (23.1%), Grand Rapids Public School newspaper (17.0%), and calendar (13.5%).

### NEMS Store Survey

The average total NEMS score for the surveyed stores was 21 with a range of 6 to 39 points (total NEMS points for a store can range from 0 to 78) (Table 4). Three of four higher scoring stores are located in the extended community area (i.e., in the 400 meters beyond the school attendance areas). In comparison to four chain grocery stores within one mile of the four neighborhood areas, all but one of these stores scored higher on the NEMS than any of the 23 stores in the four FIT neighborhoods. The average FIT neighborhood store scored well below these four larger stores on total points and in all three components of the NEMS (Table 4).

**Table 4:** NEMS Point Totals for surveyed stores.

Store ID	Availability Points (0 to 54)	Price Points (-9 to 18)	Quality Points (0 to 6)	Total Points (0 to 78)
1012	31	2	6	39
1026	32	-2	6	36
1008	28	3	-*	31
1062	20	3	5	28
1085	18	4	5	27
1036	21	0	5	26
1011	18	1	6	25
1039	17	1	6	24
1018	17	0	6	23
1070	17	0	6	23
1049	23	-1	-*	22
1064	20	-3	5	22
1007	18	-1	3	20
1016	12	2	6	20
1075	16	-2	5	19
1025	15	0	4	19
1032	14	2	1	17
1043	14	1	0	15
1099	11	-1	3	13
1078	11	-2	3	12
1051	10	2	0	12
1048	9	1	0	10
1042	6	0	0	6

Avg. for 23 Neighborhood Stores	17	0.4	3.5	21

*Larger stores*				
A	36	2	6	44
B	35	3	6	44
C	33	0	6	39
D	28	4	6	38

*Student research assistants did not record quality points for stores 1008 and 1049. It is likely that each store would have gained up to 6 more points had the quality of the fresh fruits and vegetables at each store been recorded. All four stores with zero quality points offered none of the ten identified fresh fruits or vegetables.

## Discussion

In summary, this paper described the background and rationale; study design, measurement procedures, intervention components, process evaluation procedures and baseline results of Project FIT. The baseline results indicate low physical activity, excessive screen time, low vegetable and whole grain intake, and high intake of sugar-sweetened beverages, French fries and chips and desserts - as well as a high prevalence of overweight and obesity among low income, primarily Hispanic and Black children. There are many challenges in executing and evaluating the effects of comprehensive school- and community-based interventions; this report is expected to provide useful guidance to researchers, public health professionals, and school administrators and health professionals (nurses and physical/health educators) seeking to develop similar intervention programs.

## Competing interests

The authors declare that they have no competing interests.

## Authors' contributions

JCE, KA, KAP, HJP, and JJC designed the study, established methods and questionnaires, and participated in coordination of the study. HH, TT, DK, HJO, SR, and KM participated in the day-to-day coordination and execution of the study. DH obtained funding for the study and provided general oversight to the investigators. All authors participated in the writing of the paper and provided comments on the drafts and approved the final version.

## Pre-publication history

The pre-publication history for this paper can be accessed here:

http://www.biomedcentral.com/1471-2458/11/607/prepub
